# Acromegaly and breast cancer risk: evidence from a systematic review and meta-analysis

**DOI:** 10.3389/fendo.2025.1696291

**Published:** 2025-11-10

**Authors:** Hee Christy Lee, Shruti N. Shah, John Koo, Caitlin Plovnick, Nidhi Agrawal

**Affiliations:** 1 Department of Medicine, New York University Langone Health, New York, NY, United States; 2 New York University Grossman School of Medicine, New York, NY, United States; 3 New York University, New York, NY, United States; 4 New York University Langone Health, New York, NY, United States

**Keywords:** pituitary adenoma, acromegaly, breast cancer, GH (growth hormone), (IGF-1) insulin-like growth factor I, systematic review, meta-analysis

## Abstract

**Background:**

Acromegaly is a rare endocrine disorder characterized by chronic excess growth hormone (GH) and elevated insulin-like growth factor-1 (IGF-1), which are known to have mitogenic and anti-apoptotic effects on breast tissue. While an increased risk of several malignancies has been observed in patients with acromegaly, the relationship between acromegaly and breast cancer remains unclear.

**Objective:**

To systematically evaluate the incidence and prevalence of breast cancer in patients with acromegaly and assess whether a consistent oncologic risk exists in this population.

**Methods:**

We systematically searched PubMed, EMBASE, and Web of Science from inception through early 2025 for studies reporting breast cancer in acromegaly. Citation tracking identified additional reports. After screening, 24 studies (>17,000 patients) were included, with data on cancer frequency, timing, and GH/IGF-1 levels extracted for analysis. From a subset of these studies reporting standardized incidence ratios (SIR) with 95% confidence intervals (CIs), a random-effects meta-analysis was performed to generate a pooled SIR, accounting for between-study heterogeneity.

**Results:**

This systematic review of 24 studies with diverse designs, encompassing 17,413 patients with acromegaly, found breast cancer prevalence ranging from 0.42% to 5.85%. Several studies reported elevated GH and IGF-1 levels at any cancer diagnosis, but methodological heterogeneity limited conclusions on dose–response or temporal associations. Ten studies reported SIRs with 95% CIs and were included in the pooled analysis. The pooled SIR for breast cancer among patients with acromegaly was 1.20 (95% CI: 0.94–1.54), with moderate heterogeneity (I² = 58%).

**Conclusion:**

Although there is a strong biological rationale for a link between GH/IGF-1 excess and breast cancer, current clinical studies have not shown a clear or consistently increased risk in patients with acromegaly. The mixed results likely reflect issues such as surveillance bias, differences in study designs, and limited adjustment for confounders. For now, breast cancer screening in this population should generally follow the same guidelines as the general population, with perhaps closer attention in patients who have poorly controlled disease. Moving forward, well-designed prospective studies that track cancer outcomes in relation to biochemical disease activity and control will be key to answering this question.

## 1 Introduction

Acromegaly is a rare chronic disorder characterized by excessive growth hormone (GH) secretion, most commonly due to a pituitary adenoma, which results in elevated circulating insulin-like growth factor-1 (IGF-1) levels (1). GH and IGF-1 exert mitogenic and anti-apoptotic effects on multiple tissues, including breast epithelium, and have been implicated in carcinogenesis (2–5). Preclinical studies have demonstrated that the IGF system plays a role across multiple breast cancer subtypes, with IGF-1 receptor (IGF-IR) signaling identified as a potential therapeutic target; blockade of IGF-IR has been shown to inhibit tumor growth, progression, and metastasis (6–8). Prior studies have established an increased incidence of certain cancers, including colorectal cancers, in patients with acromegaly (9, 10).

Several observational studies have examined the potential relationship between breast cancer and acromegaly, but these studies have led to inconsistent estimates of breast cancer risk in acromegaly. Variability in study design, sample size, and methodology has likely contributed to inconsistent estimates of breast cancer risk in acromegaly (11–13). Given the established role of IGF-1 in breast tissue proliferation, clarifying this potential association has important clinical implications for cancer surveillance in this population.

To the best of our knowledge, no study has systematically evaluated the relationship between acromegaly and breast cancer risk. This review focuses on observational studies examining breast cancer incidence among adults with acromegaly, a condition characterized by chronic excess of growth hormone and insulin-like growth factor-1 (IGF-1). Reported outcomes were compared with those in the general population or non-acromegaly controls, emphasizing measures such as relative or standardized incidence ratios. By synthesizing these data, our objective was to clarify whether prolonged exposure to GH and IGF-1 excess is associated with an increased risk of breast cancer in patients with acromegaly.

## 2 Methods

### 2.1 Study selection

**Figure 1:**
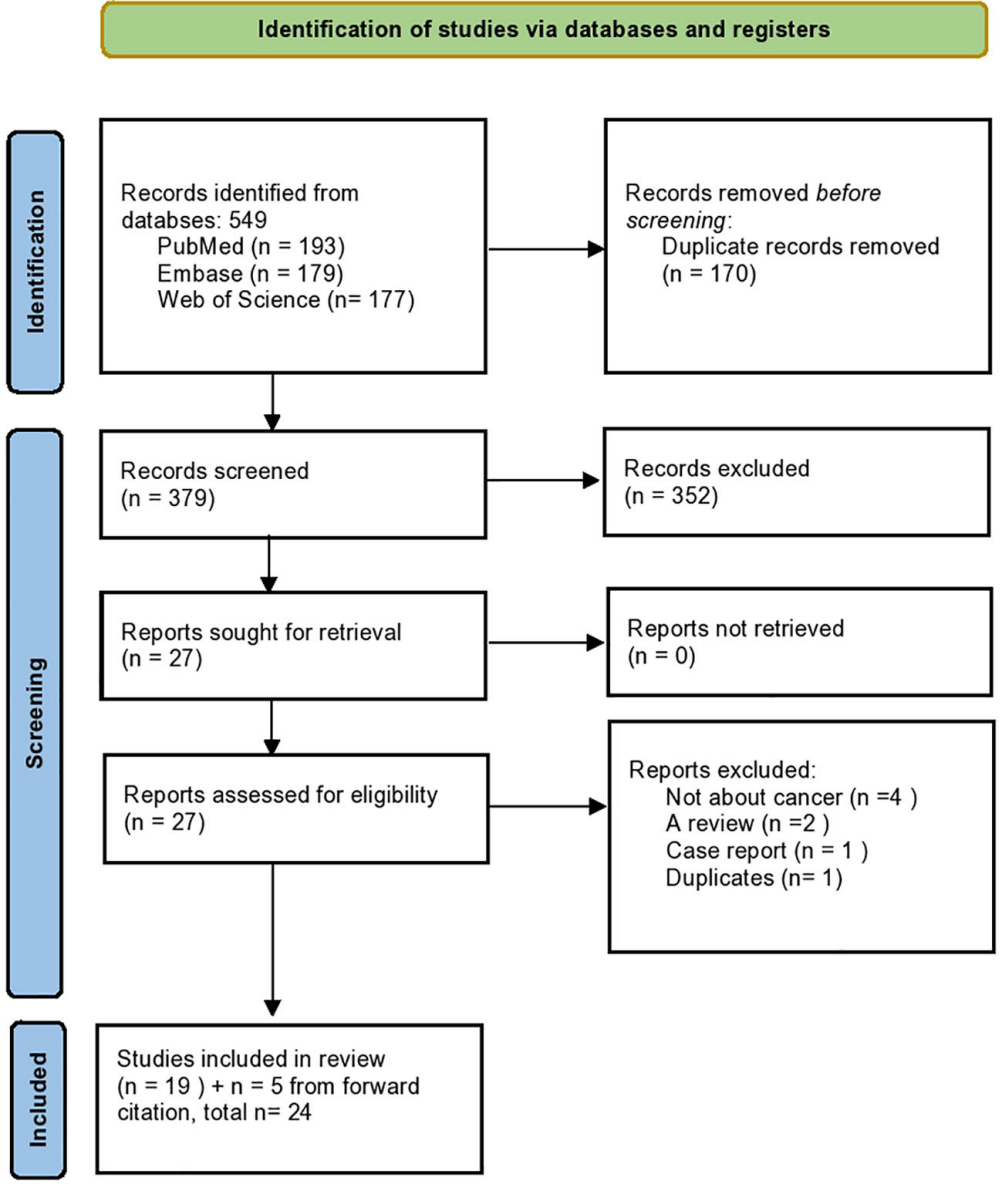
PRISMA flow diagram of study selection. Flow chart summarizing the identification, screening, eligibility, and inclusion process for studies assessing breast cancer risk in patients with acromegaly.

This systematic review was outlined to show the incidence and prevalence of breast cancer in patients with acromegaly and assess whether a consistent oncologic risk exists in this population. It was constructed by a medical librarian (CP) in consultation with the statistician (JK). Our study selection process is outlined in **Figure 1**. Further detailed information can be found in the **Supplementary Materials** under Study Selection.

### 2.2 Statistical analysis

For studies reporting standardized incidence ratios (SIR) with corresponding 95% confidence intervals (CIs), we conducted a pooled analysis to summarize breast cancer risk in patients with acromegaly. Reported SIRs and CIs were extracted directly from each publication. Observed and expected case counts were also reviewed to confirm estimates. A random-effects meta-analysis model (DerSimonian–Laird method) was applied to account for between-study heterogeneity, and results are presented as a pooled SIR with 95% CI. Heterogeneity was quantified using the I² statistic. A forest plot was generated to visually display study-specific and pooled estimates. Studies reporting effect measures other than SIR (e.g., odds ratios) were excluded from the pooled analysis but are presented descriptively in **Table 1a**.

**Table 1A:** Reported prevalence of breast cancer in acromegaly across published studies (observed cases only).

Author, year	Setting	Study design	Acromegaly patients, n	Female acromegaly patients, n (%)	Age (years)	Breast cancer cases, n (%)
*Ucan* et al.*, 2021 (*14)	Two tertiary care centers in Turkey	Retrospective cohort	280	160 (57.1%)	50.9 ± 12.1	2 (0.71%)
*Kaldrymidis* et al.*, 2016 (*19)	Single center in Greece	Retrospective, cross-sectional	110	62 (56.4%)	58.6 ± 13.8	1 (0.91%)
*Gullu* et al.*, 2010 (*20)	Tertiary care center in Turkey	Retrospective, observational cohort	105	60 (57.1%)	47.9 ± 11.5	3 (2.86%)
*Kurimoto* et al.*, 2008 (*21)	Single center in Japan	Retrospective cohort	140	86 (61.4%)	55 ± 25	4 (2.86%)
*Nachtigall* et al.*, 2020 (*22)	Multicenter in USA	Retrospective cohort	338	185 (54.7%)	n/a	4 (1.18%)^b^
*Wolinski* et al.*, 2016* (30)	Single center in Poland	Case-control	200	129 (64.5%)	53.3 ± 12.2	7 (3.5%)
*Petroff* et al.*, 2015 (*12)	National registry in Germany	Retrospective and prospective cohort	445	217 (48.8%)	58.4 ± 14.1	16 (3.6%)^b^
*Wu* et al.*, 2020 (*15)	National registry in Taiwan	Retrospective cohort	1195	591 (49.5%)	n/a	8 (0.67%)
*Popovic* et al.*, 1998 (*23)	Single center in Yugoslavia	Prospective cohort	220	137 (62.2%)	n/a	4 (1.82%)^b^
*Orme* et al.*, 1998 (*16)	Multicenter in the United Kingdom	Retrospective cohort	1239	not specified	n/a	14 (1.13%)
*Mestron* et al.*, 2004 (*24)	Multicenter registry in Spain	Voluntary report	1219	741 (60.8%)	n/a	23 (1.89%)
*Iglesias* et al.*, 2024* (47)	Single center in Spain	Retrospective cohort	544	330 (60.7%)	64.2 ± 16	9 (1.65%)^a^
*Esposito* et al.*, 2021 (*11)	National registry in Sweden	Retrospective cohort	1296	675 (52.1%)	n/a	18 (1.39%)^b^
*Durmus* et al.*, 2022 (*13)	Single center study in Turkey	Retrospective cohort	179	106 (59.2%)	53.8 ± 13.2	3 (1.68%)
*Dal* et al.*, 2018 (*10)	National registry in Denmark	Retrospective cohort	529	261 (49.3%)	n/a	9 (1.7%)^b^
*Dagdelen* et al.*, 2014 (*25)	Single center in Turkey	Retrospective cohort	160	79 (49.4%)	50.5 ± 11.5	4 (2.5%)^b^
*Cheung* et al.*, 1997 (*26)	Single center in Australia	Retrospective cohort	50	21 (42%)	55 (25–87), median (range)	2 (4%)
*Baldys-Waligorska* et al.*, 2015* (48)	Single center in Poland	Retrospective cohort	101	71 (70.3%)	51.8 ± 15.4	1 (0.99%)^b^
*Akhanli* et al.*, 2021 (*27)	Single center in Turkey	Retrospective cohort	61	61 (100%)	53 (45–59)	3 (4.92%)*
*Terzolo* et al.*, 2017* (31)	Multicenter in Italy	Retrospective cohort	1512	888 (58.7%)	n/a	24 (1.59%)^a^
*Mustacchi* et al.*, 1957 (*28)	Single center in USA	Retrospective cohort	207	95 (45.9%)	0–74, range	2 (0.97%)
*Park* et al.*, 2020 (*29)	Single center in South Korea	Retrospective cohort	718	407 (56.7%)	n/a	3 (0.42%)
*Baris* et al.*, 2002 (*17)	National registries in Sweden and Denmark	Retrospective cohort	1634	not specified	n/a	20 (1.22%)
*Freda* et al.*, 2025 (*18)	Multicenter in USA	Prospective cohort	598	289 (48.3%)	n/a	35 (5.85%)^a^

Data are presented as mean ± SD or median (IQR) unless otherwise indicated. Breast cancer prevalence (%) calculated using total acromegaly cohort which includes male and female patients unless otherwise indicated. Unless otherwise indicated, breast cancer cases were reported only in female patients. Superscripts denote exceptions:

^a^ Includes male breast cancer cases.

^b^ Sex of breast cancer cases not specified.

* Prevalence calculated from an all -female cohort.

## 3 Results

### 3.1 Study characteristics

This systematic review brought together 24 studies with a total of 17,413 patients diagnosed with acromegaly, published between 1957 to 2025. The studies varied widely in design, from early retrospective chart reviews to large registry cohorts and prospective follow-up studies. Twelve were retrospective single-center cohorts, three were multicenter retrospective cohorts, and six were registry-based or population-based cohorts. In addition, two prospective cohorts were identified, alongside one voluntary registry report and one retrospective analysis of prospectively collected data.

These studies originated from a wide range of regions with the majority from Europe (n = 16), followed by Asia (n = 3), North America (n = 2), Australia (n = 1), and two multinational collaborations. Sample sizes varied widely, ranging from as few as 50 participants in single-center cohorts to over 1,600 in large registry studies. The variation in design, setting, and cohort size—ranging from small, localized experiences to nationwide registry investigations—highlights the heterogeneity of the available evidence and contributes to the wide range of reported breast cancer prevalence in patients with acromegaly.

### 3.2 Breast cancer prevalence

Across these studies, the reported prevalence of breast cancer ranged from 0.42% to 5.85%, indicating substantial inter-study variability in detection rates (**Table 1b**). Of the included studies, 14 reported breast cancer prevalence exclusively in female patients, three provided data for both females and males, and the remaining seven did not specify the sex of the breast cancer cases. The lowest prevalence was observed in a large retrospective cohort by Park et al., in which 3 of 718 patients (0.42%) were diagnosed with breast cancer (29). In contrast, Freda et al. reported the highest prevalence, identifying 35 cases among 598 patients (5.85%) (18). Several mid-sized studies also reported rates approaching or exceeding 4%, including Akhanli et al. (3/61, 4.92%) (27) and Cheung et al. (2/50, 4.00%) (26). Additionally, breast cancer prevalence in acromegaly appeared relatively high in other mid-sized cohorts as well, reported at approximately 3–3.6% e.g., Wolinski et al. (30), Petroff et al. (12), whereas larger registry-based and multicenter studies consistently showed lower rates, generally ranging from 0.7% to 1.9% e.g., Terzolo et al. (31), Mestron et al. (24), Orme et al. (16), and Baris et al. (17).

**Table 1B:** Subset of published studies reporting standardized incidence ratios (SIRs) and odds ratios (OR) for breast cancer incidence in acromegaly.

Author, year	Setting	Study design	Acromegaly patients, n	Observed cases, n	Expected cases, n	SIR (95% CI)
*Ucan* et al.*, 2021 (*14)	Two tertiary care centers in Turkey	Retrospective cohort	280	2	3.1	0.65 (0.5-1.0)
*Petroff* et al.*, 2015 (*12)	National registry in Germany	Retrospective and prospective cohort	445	16	13.4	1.19 (0.65-1.9)*
*Wu* et al.*, 2020 (*15)	National registry in Taiwan	Retrospective cohort	1195	8	4.64	1.72 (0.86–3.44)
*Orme* et al.*, 1998 (*16)	Multicenter in the United Kingdom	Retrospective cohort	1239	14	15.09	0.93 (0.51-1.56)
*Iglesias* et al.*, 2024* (47)	Single center in Spain	Retrospective cohort	544	9; 7 female, 2 male	n/a	**6.28 (1.56-25.3)^a^**
*Esposito* et al.*, 2021 (*11)	National registry in Sweden	Retrospective cohort	1929	18	21.1	0.85 (0.5–1.3)
*Durmus* et al.*, 2022 (*13)	Single center in Turkey	Retrospective cohort	179	3	0.61	**4.92 (1.25–15.38)**
*Dal* et al.*, 2018 (*10)	National registry in Denmark	Retrospective cohort	529	9	8.1	1.1 (0.5-2.1)
*Terzolo* et al.*, 2017* (31)	Multicenter in Italy	Retrospective cohort	1512	24; 22 female, 2 male	16.8 female	1.31 (0.86-1.99)^b^
*Baris* et al.*, 2002 (*17)	National registries in Sweden and Denmark	Retrospective cohort	1634	20	15.9	1.26 (0.8-1.9)
*Freda* et al.*, 2025 (*18)	Multicenter in USA	Prospective cohort	598	35; 34 female, 1 male	21	**1.67 (1.16–2.26)**

Data are presented as mean ± SD or median (IQR) unless otherwise indicated. Statistically significant SIRs are shown in bold text.

* 95% CI approximated from figure (Petroff et al., 2015).

^a^ Reported male breast cancer risk as an odds ratio (OR) with 95% CI, not as a standardized incidence ratio (SIR).

^b^ SIR reported for female breast cancers only (male cases excluded).

To assess whether study design contributed to heterogeneity, studies were stratified into three subgroups: single-center or two center (n = 14), multicenter (n = 5), and population-based registry cohorts (n = 5). Reported breast cancer prevalence in acromegaly ranged from 0.42% to 4.92% in single-center studies (mean 2.13%), 1.13% to 5.85% in multicenter cohorts (mean 2.33%), and 0.67% to 3.60% in national registries (mean 1.72%). Despite modest numerical variation with slightly lower and narrower estimates in registries, the differences between subgroups were not statistically significant (one-way ANOVA, *p* = 0.79).

### 3.3 Standardized incidence ratios and pooled analysis

A total of 11 studies reported standardized incidence ratios (SIRs) or odds ratios for breast cancer among patients with acromegaly, with cohorts ranging from 179 to 1,929 patients (**Table 1a**). Several registry-based and population-based cohorts reported breast cancer incidence comparable to or only modestly above population rates. These included Ucan et al. (Turkey, n = 280; SIR 0.7, 95% CI 0.5–1.0), Petroff et al. (Germany, n = 445; SIR 1.19, 95% CI 0.65–1.9), Wu et al. (Taiwan, n = 1195; SIR 1.72, 95% CI 0.86–3.44), Orme et al. (UK, n = 1239; SIR 0.93, 95% CI 0.51–1.56), Esposito et al. (Sweden, n = 1929; SIR 0.9, 95% CI 0.5–1.3), Dal et al. (Denmark, n = 529; SIR 0.5, 95% CI 0.25–1.21), Terzolo et al. (Italy, n = 1512; SIR 1.31, 95% CI 0.86–1.99), and Baris et al. (Sweden/Denmark, n = 1634; SIR 1.3, 95% CI 0.8–1.9).

In contrast, single-center studies often suggested higher risk. Durmus et al. (Turkey, n = 179) reported three breast cancers compared with 0.61 expected (SIR 4.92, 95% CI 1.25–15.38). Iglesias et al. (Spain, n = 544) found nine cases (seven female, two male) and reported an odds ratio of 6.28 (95% CI 1.56–25.3). The only large prospective cohort (Freda et al., 2025, USA, n = 598) observed 35 breast cancers (34 female, one male), yielding a significantly increased SIR of 1.67 (95% CI 1.16–2.26). Overall, registry-based cohorts tended to report neutral or modestly elevated risk, whereas some single-center and prospective studies demonstrated a significant increase in breast cancer incidence.

**Figure 2:**
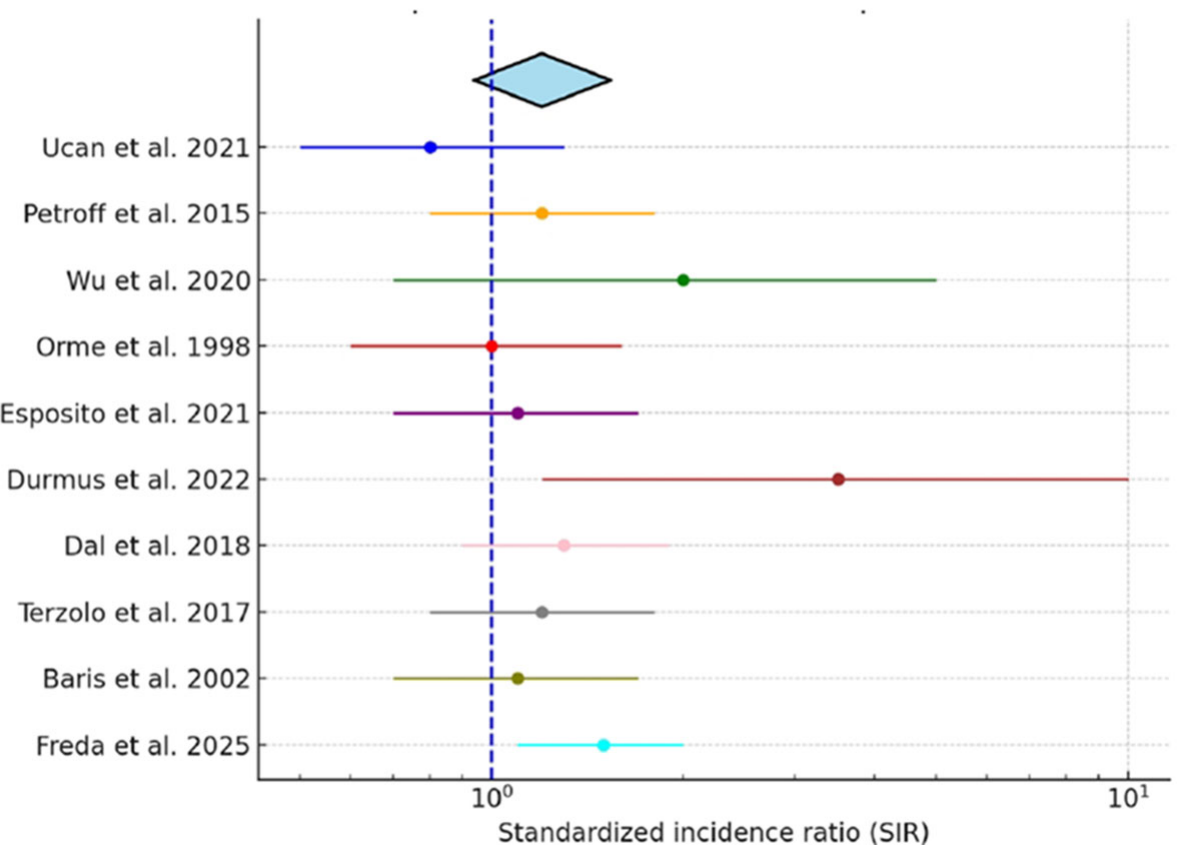
Pooled standardized incidence ratio (SIR) of breast cancer in acromegaly. Forest plot displaying individual study SIRs with 95% confidence intervals and the pooled estimate derived using a random-effects model.

In the subset of 10 studies reporting standardized incidence ratios (SIRs) with 95% confidence intervals, we performed a pooled analysis. The combined SIR was 1.20 (95% CI, 0.94–1.54), suggesting a possible increase in breast cancer risk among patients with acromegaly, although this did not reach statistical significance. Study heterogeneity was moderate (I² = 58%). A forest plot of individual and pooled SIR estimates is presented in **Figure 2**.

### 3.4 Hormonal correlates in all cancers

Furthermore, four studies provided additional data, comparing baseline GH and IGF-1 levels in acromegaly patients with and without cancer of any type (**Table 2**). Two Turkish cohorts reported no significant hormonal differences. Ucan et al. reported no meaningful difference in hormone levels, with median GH 9.5 ng/mL in patients with cancer compared to 10.4 ng/mL in those without (p=0.981), and IGF-1–738 ng/mL vs. 864 ng/mL (p=0.368). Likewise, Durmus et al. found similar results, with GH 5.5 vs. 5.5 ng/mL, (p=0.673) and IGF-1–552 vs. 646 ng/mL (p=0.91). In contrast, Dagdelen et al. observed lower IGF-1 concentrations in patients with cancer (769.1 ± 255.2 ng/mL) compared with those without (902.1 ± 276.2 ng/mL, *p* < 0.05), while GH values did not differ (22.7 vs. 22.1 ng/mL, NS). Freda et al. similarly found lower IGF-1 levels in patients with cancer (797 ± 353 ng/mL) versus those without (923 ± 385 ng/mL, *p* = 0.001), while GH did not differ significantly (10.61 vs. 10.9 ng/mL, *p* = 0.31). Overall, these findings show that reported rates of breast cancer in acromegaly vary widely, likely due to differences in study design, patient populations, and how results were reported. This variation needs to be kept in mind when interpreting the possible link, as we discussed below.

**Table 2:** Growth hormone (GH) and IGF-1 levels at diagnosis in acromegaly patients with and without any type of cancer across published studies.

Author, year	Setting	Study design	Baseline GH in patients with cancer (ng/mL)	Baseline GH in patients without cancer (ng/mL)	Baseline GH, p-value	Baseline IGF-1 in patients with cancer (ng/mL)	Baseline IGF-1 in patients without cancer (ng/mL)	Baseline IGF-1, p-value
*Ucan* et al.*, 2021 (*14)	Two tertiary centers in Turkey	Retrospective cohort	9.5 (6.4–13.4), median (IQR)	10.4 (5.3-19.7), median (IQR)	0.981	738 (594–998), median (IQR)	864 (614-1142), median (IQR)	0.368
*Durmus* et al.*, 2022 (*13)	Single center in Turkey	Retrospective cohort	5.5 (2.1–13.2)	5.5 (2.9–12.4)	0.673	552 (427–1169)	646 (470–989)	0.91
*Dagdelen* et al.*, 2014 (*25)	Single center in Turkey	Retrospective cohort	22.7 ± 29.8	22.1 ± 25.7	NS	769.1 ± 255.2	902.1 ± 276.2	**<0.05**
*Freda* et al.*, 2025 (*18)	Across New York City	Prospective, longitudinal cohort	10.61 (0.78–184)	10.9 (0.41-325)	0.31	797 ± 353	923 ± 385	**0.001**

Data are presented as mean ± SD or median (range) unless otherwise indicated. Statistically significant p-values are shown in bold text.

## 4 Discussion

Patients with acromegaly, a rare disorder caused by chronic hypersecretion of growth hormone (GH) and consequent elevation of insulin-like growth factor-1 (IGF-1), are exposed to hormones with well-established mitogenic and anti-apoptotic effects that place them at increased risk for malignancy. These hormonal imbalances drive cellular proliferation, angiogenesis, and impair DNA repair, which then can increase cancer susceptibility. Observational studies and meta-analyses have reported increased overall cancer incidence in acromegaly, particularly for colorectal cancers (4–6, 9–11). For instance, a U.S claims database study of 1,175 patients with acromegaly reported a 2.6-fold higher prevalence of malignant tumors compared with matched controls (32). This large real-world dataset captured patients across diverse care settings and included both incident and prevalent cancers. While case detection was maximized, it also increased the possibility of misclassification or coding bias. By contrast, the French Acromegaly Registry, which prospectively enrolled patients across three decades with standardized follow-up, demonstrated only a non-significant increase in standardized incidence ratios for incidental cancers, including colorectal malignancies (33).

This discrepancy suggests that while GH/IGF-1 excess may not consistently translate into a higher incidence signal at the population level, it could still play an important role in tumor biology and disease course. Down the line, they could potentially accelerate tumor growth, influence responsiveness to therapy, and or contribute to poorer survival outcomes. Indeed, recent nationwide and registry-based studies show that although overall mortality in acromegaly has declined over the past several decades, cancer has emerged as a leading cause of death, reporting cancer-related mortality as a major contributor in contemporary cohorts (10, 33, 34, 46). These findings note that the impact of GH/IGF-1 dysregulation may be more evident in cancer progression and mortality than in incidence alone, bringing out the importance of linking biological mechanisms to population-level data.

The oncogenic potential of GH/IGF-1 excess is especially pertinent to breast tissue, where several converging mechanisms may amplify carcinogenic risk. IGF-1 exerts potent mitogenic and anti-apoptotic effects, promotes angiogenesis, and activates downstream signaling cascades such as PI3K/AKT and MAPK that drive cellular proliferation. In addition, IGF-1 interacts with estrogen receptor pathways, thereby intensifying mitogenic signaling in hormone-sensitive tissues such as the breast (4, 5, 35, 36, 47). Metabolic abnormalities common in acromegaly—including insulin resistance, hyperinsulinemia, and increased sex steroid bioavailability—may further compound this risk. Consistent with these mechanistic insights, early clinical reports suggested up to a four-fold increase in breast cancer risk among women with acromegaly and more recent studies have demonstrated a positive association between cumulative GH/IGF-1 exposure and breast cancer incidence (18, 37, 38). By contrast, large registry-based cohorts such as those of Orme et al. did not detect an excess incidence compared with the general population, although disease-specific mortality was approximately 1.6-fold higher in women with acromegaly (16, 34).

Large-scale population studies outside of acromegaly consistently reinforce the role of IGF-1 in breast carcinogenesis. In a pooled analysis of 17 prospective cohorts (>4,700 cases), Key et al. reported that higher circulating IGF-1 was associated with increased breast cancer risk, particularly among premenopausal women (OR ~ 1.28; 95% CI, 1.14–1.44) (39). Using both observational and genetic instruments in ~430,000 women, Murphy et al. confirmed that genetically elevated IGF-1 was causally linked to breast cancer, independent of IGFBP-3 (IGF-binding protein-3) (OR per 5 nmol/L ≈ 1.11; 95% CI, 1.02–1.21) (35). Similarly, analyses from the UK Biobank demonstrated that higher IGF-1 was associated with increased risks for multiple cancers, including breast cancer (HR = 1.10; 95% CI, 1.07–1.14) (40). Within the EPIC cohort, Kaaks et al. reported that the association with breast cancer was strongest in younger women and in ER (Estrogen Receptor)-positive tumors (OR = 1.38; 95% CI, 1.14–1.68) (41). More recently, the EPIC-Heidelberg case-cohort study confirmed a positive association between baseline IGF-1 and breast cancer (HR 1.25; 95% CI, 1.06–1.47), while also demonstrating a U-shaped relationship between IGF-1 and mortality, with both low and high levels linked to increased cancer-related and all-cause death (42). Although these large-scale studies were conducted in the general population rather than in patients with acromegaly, they provide important external validation of the GH/IGF-1–breast cancer link, reinforcing the biological connection of an effect in acromegaly even though direct cohort evidence remains unclear (39– 41).

Despite strong biological rationale, our review of 24 studies including over 17,000 patients with acromegaly showed a wide variation in reported breast cancer prevalence, ranging from 0.42% to 5.85%. The highest rates emerged in retrospective, single-center cohorts: Freda et al. (5.85%; 35/598), Akhanli et al. (3 (4.92%; 3/61), and Cheung et al. (4.0%; 2/50) (18, 26, 27). In subgroup analyses by study design, prevalence estimates were broadly consistent across single-center, multicenter, and population-based cohorts, with registries tending to show lower means and narrower compared with single-center series and multicenter cohorts. These patterns likely reflect differences in sample size rather than true effect modification by design. Overall, study design did not explain the heterogeneity observed in the pooled analysis.

Our pooled analysis of studies reporting SIRs suggests a trend toward elevated breast cancer incidence in acromegaly, though statistical significance was not achieved. Importantly, the lower bound of the confidence interval approached 1.0, indicating that a clinically meaningful association cannot be excluded. The moderate heterogeneity reflects differences in study design and populations, which may have diluted a true effect. While the random-effects procedure we applied (DerSimonian-Laird) is a commonly applied approach in the literature, it assumes normality of the distribution of random effect; this assumption cannot be verified given the limited number of studies. Future studies with larger cohorts and standardized cancer surveillance will be essential to clarify whether the observed trend represents a causal relationship.

In Freda et al. prospective New York City cohort, 35 cases of breast were identified among 598 patients (5.85%). Patients also had markedly elevated hormone levels (median fasting GH 10.9 µg/L; mean IGF-1 923 ± 385 µg/L in those without cancer), reflecting the biochemical severity of acromegaly (18). The disproportionately high prevalence observed may reflect referral bias, greater disease severity, and more intensive surveillance in this tertiary-center cohort, which also reported elevated GH and IGF-1 at cancer diagnosis. Without matched controls or adjustment for baseline breast cancer risk factors, however, it is difficult to determine whether this represents a true biologic effect or detection bias. It was not a one-time IGF-1 level that mattered, but the long-term exposure. Over time, this persistent hormonal excess was what raised the risk of cancer. Also in this cohort, the standardized incidence ratio (SIR) for breast cancer was 1.67 compared with SEER data, pointing to a genuine excess risk beyond detection bias (18). Smaller single-center studies showed similarly high prevalence despite their limited size: Akhanli et al. observed 3 cases among 61 patients (4.92%), while Cheung et al. identified 2 cases in a cohort of 50 patients (4.0%). These findings could be from referral patterns, surveillance intensity, and statistical instability inherent to small cohorts. However, they are similar enough to reinforce the biological possibility seen in larger, prospective series (26, 27).

A recent nationwide cohort study by Mukama et al., which leveraged Swedish health registers and included more than 2,400 individuals with acromegaly without the official clinical diagnosis, further gives another perspective. The study observed a significantly increased overall cancer risk (SIR 1.3; 95% CI, 1.1–1.5) (42). In contrast to findings in Freda et al., breast cancer incidence was not elevated compared with the general population. Instead, the excess risk in Mukama et al. analysis was driven primarily by colorectal and kidney cancers which shows that broader registry-based designs may capture overall cancer susceptibility while underestimating site-specific associations such as breast cancer. Because patients with acromegaly are often followed more closely and undergo more frequent imaging and laboratory evaluations than the general population, increased surveillance can inflate cancer incidence estimates (49). Accounting for differences in screening intensity is therefore essential when interpreting reported cancer risks across studies. Nonetheless, the study reinforces the biological plausibility that has been more robustly demonstrated in larger prospective studies (18, 43).

Finally, our findings align with a comprehensive meta-analysis by Dal et al., 2018., which reported a modestly increased breast cancer risk in acromegaly (SIR 1.6; 95% CI, 1.1–2.3) (10). Combined studies from Freda et al. and the pooled results from Dal et al’s meta-analysis suggest that breast cancer risk in acromegaly if elevated, will be maybe more modest than suggested by early single-center reports. Still, biochemical profiles, and population-based IGF-1 data consistently reinforce the biologic plausibility that GH/IGF-1 excess contributes to breast carcinogenesis.

These findings should be interpreted with caution, as several important limitations exist. First, the included studies differed widely in their design, sample size, and methods of data collection. Many were retrospective single-center case series with relatively small sample sizes, while others were registry-based national cohorts with more standardized data capture but often limited clinical detail, even though limitation for heterogeneity was attempted to be addressed by performing a sub-analysis by study design. Second, there was a lack of information on how breast cancers were detected. Because most studies did not specify whether cancers were identified through routine screening or clinical presentation, it is unclear to what extent detection method influenced the reported incidence and prevalence estimates.

Furthermore, the majority of studies did not clearly report whether breast cancers occurred before or after the diagnosis of acromegaly, making it difficult to interpret the timing of events. This is important because acromegaly is often diagnosed only after years of unrecognized disease activity (44). Therefore, some cancers reported as occurring ‘before diagnosis’ may have actually developed during a long period of undetected GH and IGF-1 excess. This delay can blur the distinction between pre- and post-diagnosis cancers and may lead to inconsistent reporting across studies. In our review, such differences likely contributed to some of the variability seen in incidence and prevalence estimates (48). Future studies should clearly describe the timing of cancer diagnosis relative to both symptom onset and biochemical confirmation of acromegaly, and take diagnostic delay into account when assessing cancer risk.

Another factor that may influence breast cancer risk in acromegaly is gonadal status and exposure to estrogen–progestin therapy. However, most studies did not report on the prevalence of gonadotropin deficiency or the use of hormone replacement therapy, limiting the ability to assess their potential impact on cancer risk (45). Similarly, data on male patients were also scarce. Breast cancer in men with acromegaly may be underrecognized—particularly in those with hypogonadism-related gynecomastia—since routine breast imaging is rarely performed in this population. Future studies should include both sexes and apply systematic screening to better define sex-specific cancer risks in acromegaly. Without these adjustments, it is difficult to tease out the independent effect of acromegaly or GH/IGF-1 excess on breast cancer risk.

This review shows that the link between acromegaly and breast cancer is complex and still unclear. The GH/IGF-1 axis makes the connection biologically plausible, but clinical studies have been inconsistent. Reported prevalence rates vary widely, and only a few studies provide statistically significant SIR estimates. The strongest signals come from cohorts with detailed hormonal data and pooled analyses, while registry-based studies have often shown no clear excess, likely due to methodological limitations. For now, we cannot say there is definitive causal relationship. Nonetheless, maintaining hormonal control and following established cancer screening guidelines remain essential for care. Future prospective studies will be essential to determine whether biochemical control affects breast cancer risk in this population.

## Data Availability

The original contributions presented in the study are included in the article/**Supplementary Material**. Further inquiries can be directed to the corresponding author.
