# Supplementary material for: Acromegaly and breast cancer risk: evidence from a systematic review and meta-analysis
Source: Front Endocrinol (Lausanne). 2025 Nov 10;16:1696291. doi: 10.3389/fendo.2025.1696291 (PMC12640837; doi:10.3389/fendo.2025.1696291)
Supplement: Supplementary file 1 [file DataSheet1.docx]

**Search Strategy:**

A comprehensive literature search was conducted across PubMed, Embase, and Web of Science (SCI-EXPANDED) from inception to March 26, 2025. The search combined terms related to *breast cancer* (e.g., “breast neoplasms,” “mammary carcinoma”) and *acromegaly* (e.g., “acromegaly,” “GH-secreting adenoma,” “growth hormone hypersecretion”), using both keywords and controlled vocabulary (e.g., MeSH, Emtree). Filters were applied to include only English-language articles and peer-reviewed original or review articles.

- PubMed: 232 results → 193 after English language filter
- Embase: 196 results → 179 after English language filter
- Web of Science: 187 results → 181 (English) → 177 (articles/reviews)

Detailed search terms are available upon request or in the Supplementary Appendix.

PubMed:

("Breast*"[tw] OR "Breast"[Mesh] OR "breast neoplasms"[mesh] OR "mammary cancer*"[tw] OR "mammary neoplasm*"[tw] OR "mammary carcinoma*"[tw]) AND ("acromegaly"[MeSH Terms] OR "acromegaly"[tw] OR "acromegalies"[tw] OR "growth hormone-secreting pituitary adenoma"[tw] OR "somatotroph adenoma"[tw] OR "growth hormone producing"[tw] OR "growth hormone hypersecretion"[tw] OR "GH secreting"[tw] OR "GH producing"[tw] OR "GH hypersecretion"[tw]) 232 -> English only 193

 Embase:

1. (breast*.mp. or exp breast/ or exp "breast tumor"/ or "mammary cancer*".mp. or "mammary neoplasm*".mp. or "mammary carcinoma*".mp.) and (exp acromegaly/ or "acromegaly".mp. or "acromegalies".mp. or "growth hormone-secreting pituitary adenoma".mp. or "somatotroph adenoma".mp. or "growth hormone producing".mp. or "growth hormone hypersecretion".mp. or "GH secreting".mp. or "GH producing".mp. or "GH hypersecretion".mp.)
2. Limit 1 to article
3. 196 -> English only 179

 Web of Science: Science Citation Index Expanded (SCI-EXPANDED):

("acromegaly" OR "acromegalies" OR "growth hormone-secreting pituitary adenoma" OR "somatotroph adenoma" OR "growth hormone producing" OR "growth hormone hypersecretion" OR "GH secreting" OR "GH producing" OR "GH hypersecretion") AND (Breast* OR "mammary cancer*" OR "mammary neoplasm*" OR "mammary carcinoma*")

187 -> English only 181 -> article/review article only 177

 549 total -> 379 when duplicates removed.

**Study Selection:**

After completing the search strategy, two authors independently reviewed 379 titles and abstracts to determine eligibility. Studies were excluded if they were case reports, comparative studies, reviews, animal studies, duplicates, or not published in English. Full texts of potentially relevant articles were then assessed in detail. Any differences in opinion were discussed until consensus was reached, and when necessary, a third author provided input. In addition to the database search, the authors manually screened the reference lists of included papers and relevant reviews to identify additional studies. This backward and forward citation tracking yielded five more eligible articles, which underwent the same screening and quality assessment process. In total, 24 studies met the inclusion criteria and were included in the final review.
